# Emergency department clinical leads’ experiences of implementing primary care services where GPs work in or alongside emergency departments in the UK: a qualitative study

**DOI:** 10.1186/s12873-020-00358-3

**Published:** 2020-08-14

**Authors:** Michelle Edwards, Alison Cooper, Freya Davies, Rebecca Sherlock, Andrew Carson-Stevens, Delyth Price, Alison Porter, Bridie Evans, Saiful Islam, Helen Snooks, Pippa Anderson, Aloysius Niroshan Siriwardena, Peter Hibbert, Thomas Hughes, Matthew Cooke, Jeremy Dale, Adrian Edwards

**Affiliations:** 1 Division of Population Medicine, Cardiff University, Heath Park, Cardiff, UK; 2 Swansea University Medical School, Swansea University, Swansea, UK; 3 Swansea Centre for Health Economics, College of Human and Health Sciences, Swansea University, Swansea, UK; 4 Community and Health Research Unit, School of Health & Social Care, University of Lincoln, Lincoln, UK; 5 Centre for Healthcare Resilience and Implementation Science, Australian Institute of Health Innovation, Macquarie University, Macquarie Park, Australia; 6 Emergency Department, John Radcliffe Hospital, Oxford, UK; 7 Warwick Medical School, University of Warwick, Coventry, UK; 8 Academic Primary Care, Warwick University, Coventry, UK

**Keywords:** Emergency care, Urgent care, General practitioners

## Abstract

**Background:**

To manage increasing demand for emergency and unscheduled care NHS England policy has promoted services in which patients presenting to Emergency Departments (EDs) with non-urgent problems are directed to general practitioners (GPs) and other primary care clinicians working within or alongside emergency departments. However, the ways that hospitals have implemented primary care services in EDs are varied. The aim of this study was to describe ED clinical leads’ experiences of implementing and delivering ‘primary care services’ and ‘emergency medicine services’ where GPs were integrated into the ED team.

**Methods:**

We conducted interviews with ED clinical leads in England (*n* = 19) and Wales (*n* = 2). We used framework analysis to analyse interview transcripts and explore differences across ‘primary care services’, ‘emergency medicine services’ and emergency departments without primary care services.

**Results:**

In EDs with separate primary care services, success was reported when having a *distinct* workforce of primary care clinicians, who improved waiting times and flow by seeing primary care-type patients in a timely way, using fewer investigations, and enabling ED doctors to focus on more acutely unwell patients. Some challenges were: trying to align their service with the policy guidance, inconsistent demand for primary care, accessible community primary care services, difficulties in recruiting GPs, lack of funding, difficulties in agreeing governance protocols and establishing effective streaming pathways. Where GPs were integrated into an ED workforce success was reported as managing the demand for both emergency and primary care and reducing admissions.

**Conclusions:**

Introducing a policy advocating a preferred model of service to address primary care demand was not useful for all emergency departments. To support successful and sustainable primary care services in or alongside EDs, policy makers and commissioners should consider varied ways that GPs can be employed to manage variation in local demand and also local contextual factors such as the ability to recruit and retain GPs, sustainable funding, clear governance frameworks, training, support and guidance for all staff. Whether or not streaming to a separate primary care service is useful also depended on the level of primary care demand.

## Background

To manage increasing demand for emergency and unscheduled care NHS (National Health Service) England policy has promoted services in which patients presenting to type 1 EDs (consultant led 24-h services with full resuscitation facilities) with non-urgent problems are directed to general practitioners [1]. In 2017, NHS England allocated £100 m ($127 m) of capital funding to enable primary care services to be implemented in or alongside EDs. The aim was to direct unscheduled primary care type patients presenting to EDs to general practitioners (GPs) or other primary care clinicians working in a distinct service, based on clinical criteria, and with a robust governance structure in place. The recommended NHS service model was based on the model at Luton and Dunstable Hospital (Bedfordshire, England) whereby patients attending the emergency department have a brief initial assessment at the ‘front door’ by emergency department nurses and if found to have non-urgent problems are ‘streamed’ to primary care clinicians working in a co-located but distinct primary care service [2].However, the ways that hospitals have implemented GPs in their EDs are more varied [3] and there are few data about the impact of using primary care clinicians in or alongside EDs [4–8]. There are concerns about negative effects, including that primary care services located in or alongside EDs may encourage more patients to attend (‘provider-induced demand’) [1, 9, 10]. In Wales there was no policy drive or funding initiatives to have primary care services in or alongside emergency departments. However, some Welsh hospitals have had experience of employing GPs within their EDs, working in various roles.

We recently proposed a taxonomy to describe the form and function of primary care models in and alongside EDs [3]. We proposed that EDs with primary care models function on a spectrum of integration, from GPs fully integrated in an ‘emergency medicine service’ to GPs working in a separate ‘primary care service’. We also described how some services might operate with some characteristics across the spectrum in terms of various individual, department level or wider system level factors [3].

This study describes the experiences of ED clinical leads in managing the delivery of emergency care services with and without primary care services in or alongside them. Whilst a range of clinicians may be employed in these primary care services (e.g. GPs, nurse practitioners, and paramedics),our focus is on the use of GPs, as this has been the policy focus to date [2].

## Method

### Survey sample

In the UK there are three types of emergency departments: type 1 consultant led 24-h services with full resuscitation facilities);type 2 are consultant led facilities for specialities (e.g. for treating eye problems or dental problems); and type 3 departments are for treating minor injuries or minor illness [11, 12]. Type 1 emergency department attendances accounted for 63% of attendances in 2018/2019 [13]. Whilst GPs often work in type 3 emergency departments, recent policy was focused on more use of GPs in type 1 emergency departments to manage an increasing demand for primary care type problems in this setting. This study was a preliminary stage in a larger evaluation of GPs working in type 1 emergency departments that was commissioned by the National Institute of Health Research (NIHR) Health Service and Delivery Research Programme (HS&DR) [3]. We focused on ED clinical leads (generally also termed “clinical directors”) as key informants because of their role in managing recruitment and staffing within the department and their overall accountability for the quality of care in the department.

In September 2017 we developed a survey and invited clinical leads of all 185 type 1 EDs [11] in England and Wales to complete an online survey (www.onlinesurveys.co.uk) to obtain national-level information about the ways that GPs work in or alongside the EDs (excluding out of hours-primary care services) [11]. Survey topics included: location of GPs; types of patient groups; use of investigations; funding and governance arrangements; aims of the service; enablers and barriers to setting up the service and service changes [3].

Seventy-seven respondents (40%) completed the survey and agreed to be contacted for a follow-up telephone interview. Most surveys were completed by clinical directors, with some completed by medical directors and some by ED consultants. We also obtained data from a further 41 English departments from additional data sources (e.g. Royal College of Emergency Medicine), increasing our coverage of how services operate to 62% (*n* = 118/189) of Type 1 emergency departments in England and Wales. We know from all our data sources that a high percentage of EDs had applied for capital funding to improve their existing service or implement a new service design and that this did not differ between respondents (82%) and non-respondents to the survey (84%) [3].

### Qualitative interview sample

Survey responses and additional data were screened by three researchers [ME, AC, RS] and a purposive sample of 30 potential participants was identified from the 77 survey respondents (see Table 1 below); nine declined to take part or did not respond to invitations.

**Table 1:** Selection criteria for the purposive sample of Emergency Departments suitable for interview

Implementation	Service implemented since 2010
**Primary care service models**	Delivered one of three models of ED services: separate primary care services *[inside or outside the footprint of the ED]*, integrated emergency medicine services, and no GPs used in the ED
**Enablers/barriers to set up**	Able to discuss enablers/barriers to setting up and delivering the primary care service
**Types of patients**	Responses indicating a range of types of patients seen
**Investigations available to GP**	Responses indicating variable extent of investigations and interventions to which GPs had access
**Hospital context**	Variety of contexts -including hospitals in rural and urban locations/towns, small and large hospitals, higher vs lower attendances
**Location**	Spread of geographical location in England and Wales

### Interview guide

We developed semi-structured interview guides specifically for this study and included a core set of topics: the aims of the primary care service; enablers and barriers to implementing and delivering a service; GPs’ roles; streaming; patient demand and flow. Tailored questions were also included to explore comments made in survey responses. Interview guides were piloted with two clinical leads and refinements were made to some of the questions throughout the interview period (March 2018 to March 2019) to explore nuances in primary care models. However, data saturation was examined and achieved and at the end of the interview period, all themes had been explored and all participants had answered questions on the core set of topics and further questions relevant to the model of service and challenges they described in their survey response (see additional file 1).

### Data collection and analysis

We conducted interviews with 21 emergency department clinical leads (18 were clinical directors at the time of the interview and 3 had previously been clinical leads and were now medical directors – higher level administrative responsibility), 18 by telephone and three in person. All were audio-recorded, with participants’ consent, and transcribed verbatim. An initial thematic coding framework was created in NVivo 11 (QSR International, Daresbury) based on the research questions and the survey responses. Interview transcripts were coded to themes/subthemes within the thematic framework by ME, also allowing for new themes to be identified [14]. When it was established that there were no additional concepts observed then sampling was terminated.

A proportion of the transcripts (40%) was independently coded by a second author (DP). Agreement between coders was high (> 90%), with only minor amendments and clarifications made to the coding. The themes were then mapped to service function to identify patterns of commonality and differences between and within models [15].

### Patient and public involvement

Patients and public members were involved in the design of this study and used their experience of being an NHS patient to contribute to the content of the questionnaire and qualitative interview guides. They will be involved in disseminating the study results to wider patient communities by developing, planning and delivering presentations for patient groups and advising on the content and format of presentations to stakeholder groups (commissioners, policy makers, practitioners and academics).

## Results

We first present the sample characteristics of participants selected from the online survey and then themes identified in the qualitative data.

### Sample characteristics

A sample of participants from 21 EDs was included and thematic saturation was achieved. Service models were categorised as either primarily integrated with the emergency medicine service or a separate primary care service (see Table 2). Nineteen EDs were in England, 11 had GPs working in separate primary care services (seven “inside” ED footprint and four “outside” the ED footprint [3]), five EDs had GPs integrated in the ED (“inside-integrated” models [3]) and three EDs did not currently employ GPs. Although some hospitals fitted mainly into one category, some described features associated with both types of service. There were no primary care models reported by survey respondents in Wales. Two were included as examples of departments currently without GPs, but each had prior experience of using GPs occasionally.

**Table 2:** Models of service

Model of Service	Number of hospitals
Separate primary care service	Inside ED footprint 7
	Outside ED footprint 4
Integrated emergency medicine service	5
No current Primary Care service	5

### Themes in the data

Three main themes were evident from the qualitative interview data:

- *Achieving the aims of implementing and delivering separate primary care services or integrated emergency medicine services*
- *Challenges in implementing and delivering primary care services and emergency medicine services*
- *Facilitators and barriers to primary care streaming*

#### Achieving the aims of implementing and delivering separate primary care services or integrated emergency medicine services (see Table 3 for example data)

##### Separate primary care services

Separate primary care services were implemented with the aim of reducing waiting times and improving flow, by streaming patients identified to have primary care needs away from the main area of the ED. These included urgent treatment centres, primary care “walk-in” centres or treatment rooms located either *inside the footprint of the ED* (hospitals 4, 5,6,7,8, 17, 20) or *elsewhere on the hospital site* (hospitals 10, 11, 13, 18). Some participants described how they aimed to replicate the Luton and Dunstable (L&D) model (promoted in NHS policy) (hospitals 5, 15, 18 and 20).

**Table 3:** Reported successes in meeting the aims of each service

Type of service	Aims/ perceived benefits	Quotes
**Separate primary care services**	Focusing GPs on primary care patients without access to investigations improves flow	“We decided a long time ago that he walk-in centre would do no investigations, so they don’t have access to x-ray, and they don’t have access to blood tests. Because we wanted them to have a quick flow, and that doing investigations slow them down”. (Hospital 11)
	Focusing GPs on primary care patients frees up ED staff to focus on more acutely unwell patients	“Partly it’s about having another pair of hands, another staff member, but it’s also about freeing up ED staff and their skills to see the more injured or acute end of the spectrum, and letting GPs see their appropriate patients”. (Hospital17)
**Integrated emergency medicine services**	Shared governance structure enables load balancing – the ability to share patient lists	“It might be that there’s only one or two patients in the Urgent Care stream and there’s ten in the Emergency Care stream, so again because we all work in one hub essentially and we’re all under one governance hat, a GP can flip streams if there’s something appropriate in the stream next door”. (Hospital 3)
	Multidisciplinary team with a wider range of skills to manage demand	“The big bonus of it is it gives us a bigger staff network to use, to be open and honest with you, it also shares ideas” (Hospital 13)
	Opportunities for shared learning	“We’ve learnt from our GPs here as well as them learning from us. I think we learn a lot from the GPs here in terms of assessing risk”. (Hospital 9)
	Adding more value to the cost of employing GPs	“So there’s a lot of non-silo working. I’m not wanting someone being paid good money sitting in a room doing nothing”. (Hospital 13)
	Using a GP as an autonomous Decision maker vs information collector	“These are fully qualified GPs, they’re senior decision makers, they’re autonomous, they’re not coming back to ask you information, they’re not coming back to ask how to manage patients all the time”. (Hospital 3)

As part of the official NHS model, GPs were expected to see only primary care patients, without accessing hospital investigations (hospitals 7, 11, 13, 18). However, in two EDs there was flexibility for some GPs to see patients with minor injuries (hospitals 15, 17). Some of the aims achieved in relation to GPs working in a separate service were reported as reduced waiting times and improved flow in the ED, enabling primary care patients to be seen quickly without investigations, and enabling ED doctors to focus on more acutely unwell patients (hospitals 4, 6,7,10,11,13,17).

##### Integrated emergency medicine services

Participants who managed integrated emergency medicine services recognised that the nature of demand in the ED is varied and does not necessarily fit into clear cut definitions of primary care or emergency care, that GPs have skills, experience and special interests that can be used to manage this variation. GPs in these services were recognised as autonomous decision-makers. The aims of such services were to: (1) improve waiting times and flow by focusing on primary care patients and sometimes also patients with minor injury or more acutely unwell patients (hospital 3); (2) to reduce admissions by focusing on frail/elderly patients (hospitals 14, 21) or (3) to work in the ED as a middle grade ED doctor seeing undifferentiated patients (if experienced in emergency care) to fill gaps in ED staff recruitment (hospital 9).Hence, GPs’ caseloads sometimes included a wide range of acuity and primary care streaming was not always used (hospitals 9, 14, 21).

Participants reported several advantages to integrating GPs in the ED: it gave them a multidisciplinary team of staff with a range of skills to manage the demand; it created opportunities for sharing advice and learning between GPs and ED clinicians; it also provided an opportunity to gain more value for the cost of employing GPs. Having an overarching governance structure enabled patient lists to be shared and for some GPs to see emergency care patients and hence have greater effect on ED performance (hospitals 3, 9, 14, and 21).

##### Services with characteristics of both types of service

Some EDs functioned with characteristics of a separate service and an integrated service, depending on what they wanted to achieve. In hospital 3,GPs mainly saw primary care patients in rooms in a separate corridor (as described in the NHS policy guidance), but some also worked collaboratively in a multidisciplinary team to see emergency care patients. At hospitals 14 and 21, GPs focused on different patients depending on the time of day –during daytime hours they saw frail elderly patients in the ED to help reduce admissions but during the late afternoon and evening they saw primary care patients because the demand for primary care was greater then.

### Challenges in implementing and delivering primary care services and emergency medicine services (see Table 3 for example data)

#### Low demand for primary care

Some participants reported a low demand from patients presenting with primary care type problems, attributed to local demographics (e.g. older age people living in the area) or easily accessible and good quality community-based primary care and ambulatory care services (hospitals 4, 5, 15, 19). Some participants reported that their primary care service was not cost-effective due to the high costs of GPs, low demand and little or no impact on waiting times, flow or use of investigations. Consequently, some primary care services were scaled-down (hospitals 4, 5, 9, 18) or discontinued (hospitals 12, 14, 19). Some participants commented that the Luton and Dunstable model was not appropriate for their service (hospitals 5, 18).

#### Provider-induced demand

Participants expressed concerns that implementing a primary care service might cause ‘provider-induced demand’ [9] (hospitals 3, 6, 7). Two hospitals limited the visibility of their primary care service by ensuring there was no media publicity (hospitals 3,7) and not having public-facing signs indicating GPs were working within the department(hospital 3). Conversely, hospital 6 had a primary care walk-in centre located inside a new, widely publicised ED, and reported additional demand from patients across the region.

#### GP staffing

Participants reported frequent rota gaps due to difficulties recruiting GPs (hospitals 1, 4, 5, 7, and 8), or attracting GPs to work early, late or weekend shifts or over holiday periods (hospitals 4, 7, 8).Sometimes employing locum GPs from out-of-area increased costs to an unsustainable level and contributed to problems providing consistent rota cover (hospitals 5, 7). In hospital5 the inability to recruit local GPs and a lack of funding to employ locum GPs (and a streaming nurse) meant that middle grade doctors and primary care nurse practitioners saw primary care patients in a part of the ED that was built for the intended primary care service.

Lower than expected primary care demand and budgeting constraints meant that some hospitals reduced their number of GPs, limiting the capacity to stream all primary care patients away from the ED and leaving no contingency for staff absence. This often led to closing the service unexpectedly and the primary care patients being seen in the ED (hospital 18).However, participants where GPs were included in integrated emergency medicine services did not report challenges with recruiting GPs (hospitals 3, 8, 14, 19, 21). In hospital 3 there were18 GPs employed, and weekend working was included in their contracts to ensure consistent cover. The ability to offer an NHS contract and cover indemnity costs helped some services recruit GPs (hospitals 4, 11).

#### Factors preventing some hospitals from implementing a service

Some hospitals did not implement a service because of: lack of space for GPs and a potential reduction in ED space which might negatively impact ED flow(hospital 16); competing funding priorities for extended community primary care services (hospital 15); and previously unsuccessful pilot services (hospitals 12, 15).Some participants were concerned that if GPs worked inside the ED they might be tempted to work outside the role expected of them and see emergency care patients, potentially leading to quality and safety concerns (hospitals 2, 16).

In Wales there were no policy or funding initiatives for primary care services in EDs; two departments had ED staff with previous experience of working as GPs but were employed in a middle grade ED doctor role. These hospitals had previously piloted models with GPs to focus on primary care type patients but there was no longer funding or available GPs (hospitals 1, 2).

### Facilitators and barriers to primary care streaming (see Table 4 for example data)

Various methods of primary care streaming were used: patients were sometimes allocated to a primary care stream after a brief *streaming assessment* by a nurse in an area near the front door before booking in at ED reception and being streamed to a primary care service (hospitals 7, 8, 11, 13); or during a *triage assessment* (hospitals3, 4,6, 14, 15, 19) after being booked in by a receptionist and before being streamed to a primary care service. Some departments only used a triage process (hospitals 5, 9, 14, 21) and GPs selected patients to see according to specific guidance or that they felt were appropriate for their skill sets.

**Table 4:** Reported challenges in implementing and delivering primary care services and emergency medicine services

Type of service	Challenges	Quotes
**Separate primary care service**	Low and inconsistent primary care demand	“Do we have enough patients to keep the GPs busy, probably we don’t, so we’re seeing just over 2 patients per hour, on average, and it also depends on if it’s a busy shift where there’s lots of appropriate patients”. (hospital 4)
	Difficulty in recruiting GPs and covering the rota	“So we started to employ, or rather the CCG employed, GPs to do an early and a late shift Monday to Friday in the department. They were never successful at fully recruiting to cover all those slots”. (Hospital 8)
	Inability to provide a consistent service	“Some days it doesn’t open at all because someone’s off sick and they can’t cover it last minute”. (Hospital 18)
**Integrated emergency medicine service**	Low primary care demand	“The CCG has terminated that because they felt that they wanted them to be seeing 3 to 4 an hour, and we just couldn’t give them the patients, we just didn’t have the right kind of patients for them to see”. (Hospital 19)
	Not labelling the primary care area in an integrated model	“We’ve not changed the label outside the hospital, it doesn’t say Urgent Care Centre, it doesn’t say anything else because we didn’t want to have a honey-pot effect of attracting more people in” (Hospital3)
	Avoiding publicity to manage provider induced demand	“We kind of opened it surreptitiously, we’ve never opened with a big bang, so I think any increase in demand has been via 111 rather than walk-un patients” (Hospital 7)
**No primary care provision**	Lack of space in the ED for GPs	“I think if we had, from a pragmatic point of view, a GP in the department, it would increase pressures because by definition of taking up a room, to deliver that service, that would be one less room to flow patients through from an ED perspective”. (Hospital 16)
	Insufficient funding and inability to recruit	“That’s always been our difficulty I think, in recruitment, is we can’t pay anything like GPs would have been paid to work through OOH”. (Hospital 1)
	Concern that GPs ‘go native’ i.e. start behaving like ED clinicians and ordering lots of tests.	“My worry is that once in the ED footprint, and working that closely with the ED teams, is how soon before they sort of fall back into a non-primary care role”. (Hospital 16)

#### Definitions of streaming and triage [3]

Streaming assessment - an operational activity to direct low acuity patients to an appropriate clinician based on clinical availability and suitability.

Triage assessment- a clinical activity to sort patients by acuity so that those with the greater need are seen first.

#### Facilitators of streaming

(see Table 5) Some participants reported that primary care streaming reduced the number of patients in the ED, contributing to overall improvements in waiting times and flow (hospitals 3, 7, 20). Factors reported to facilitate effective primary care streaming were: consistent demand from primary care patients; experienced and confident nurses; clear guidelines (hospitals 3, 6, 10, 11, 13, 16, 20); NHS or shared clinical governance (hospital 3); training, evaluation and improvement workshops (hospitals 6, 20). Mentoring and support from senior ED staff also improved streaming (hospitals 4, 10, and 16). In some departments senior ED clinicians monitored and supervised streaming, sometimes moving patients from an emergency care to an urgent care stream (hospitals 3, 4, 15,20).

**Table 5:** Reported facilitators and barriers of primary care streaming

Type of Service in ED	Facilitators	Quote
**Separate primary care service**	The ability to stream children to a GP	“Since the Urgent Care centre (UCC) opened up, we saw a 50% reduction in children being seen in the ED, because they were being streamed to the UCC rather than us”. (Hospital 7)
	Streaming guidance and support	“We’ve developed our own guidelines, I mean based on the facility itself, as to who should go where. Because all the staff - apart from the GPs who are in the Urgent Care centre - are our staff, it kind of works, because people will speak to each other as well, and ask where they think someone is appropriate for”. (Hospital 10)
	Senior clinician monitoring of streaming	“Sometimes the doctor in charge will have a look through the box and notice, on reviewing the triage notes, will think ‘actually, that sounds very suitable for primary care’, and sometimes the primary care physician themselves will have a look through the box because they will not have anyone to see, and so they’ll identify ones that they can see”. (Hospital 4)
	**Barriers**	**Quote**
	Lack of shared governance and poorly established working relationships	“so there wasn’t a team of doctors that owned the GP service, and we didn’t get to know them, and they didn’t get to know us, and so there are points of tension where the streaming nurses have sent patients across and they’ve been sent back,” (Hospital 18).
	Lack of shared governance- reduces collaboration	“My impression was that the company who owns the service would only allow the GP to see a restricted range of patients because of Governance”. (hospital 7)
	Insufficient primary care demand	“We tried using Luton streaming model, but using Luton streaming model we ended up with less than 2 patients an hour going to them”. (Hospital 19)
**Integrated emergency medicine service**	**Facilitators**	**Quote**
	Shared clinical governance encourages collaboration and enables flexibility in which patients GPs can see	“Because we all work in one hub essentially and we’re all under one governance hat, a GP can flip streams if there’s something appropriate in the stream next door”. (Hospital 3)

#### Barriers to streaming

(see Table 5) One of the main barriers to primary care streaming related to establishing governance pathways and policies and reaching an agreement on which types of patients a GP should see(hospitals 4, 7, 18). Poor working relationships between staff working in an NHS commissioned ED service and a primary care service operated by an independent primary contractor meant that reaching agreement on initial assessment time targets and which patients GPs should see was challenging. Sometimes ED staff had to see patients waiting to be seen in the primary care service to ensure assessment time targets were achieved (hospital 7), or patients streamed from the ED to the primary care service were sent back to the ED because they did not meet the criteria set out by the primary care service (hospital 18). Two participants reported that setting up streaming was very challenging where primary care demand was low (hospitals 4 and 14). Despite internal evaluations and quality improvement efforts to improve primary care streaming the numbers of patients considered suitable for primary care was too low at one ED so the primary care service was withdrawn (hospital 14).

## Discussion

### Principal findings

In EDs with separate primary care services, participants reported success in terms of having a *distinct* workforce of primary care clinicians, who improved waiting times and flow by seeing primary care type patients in a timely way, using fewer investigations, and enabling ED doctors to focus on more acutely unwell patients. However, trying to align their service with the policy guidance was reported as challenging because of inconsistent demand for primary care, if there were good quality and accessible community primary care services, difficulties in recruiting GPs, lack of funding, difficulties in agreeing governance protocols and establishing effective streaming pathways.

Where GPs were integrated into their ED workforce, GPs saw primary and emergency care patient’s or focused on specific patient groups (e.g. frail elderly) but not the sickest patients requiring resuscitation. Success was reported in terms of managing the demand for both emergency and primary care and reducing admissions. The challenges of recruitment and rota coverage less prominent in this type of service. Only one ED operated streaming within their triage process. Single governance arrangements in the integrated emergency medicine services were reported more favourably than multiple governance arrangements in the separate services.

#### Strengths and limitations

We used data from a national survey to purposively identify EDs with different characteristics and varying experiences that could be followed up with in-depth qualitative interviews. Our response rate to the survey of 40% may have introduced a response bias. However, we compared the use of GPs in our respondents and non-respondents (including some additional data from Royal College of Emergency Medicine [3]) and found no evidence of different GP models, or of non-response bias in terms of applications for streaming capital funding (or not) in England in 2017. Over 80% of departments applied for capital funding to improve or implement a primary care service so the drive to fit with the policy was widespread across England and our sample reflected this. Our sample included a range of different ways that emergency services with primary care staff are configured. However, there may be other characteristics influencing how primary care services function in EDs that were not included in the sampling frame for this study. Other countries also may also have different service models [16].

The findings reflect the views and experiences of the senior clinical lead interviewed at each participating service and the views of other staff and stakeholders within these services may differ from those included here. Furthermore, measures of success were self-reported and we did not gather quantitative data about reduced waiting times or improved flow. We did not explore in detail the challenges with financing primary care services within these interviews.

#### Previous research

Other authors have suggested that for primary care models in or alongside emergency departments to be successful and sustainable they need to be distinct primary care services with GPs seeing low acuity patients [10]. Whilst we identified evidence supporting these model types, we also identified challenges such as: perceived low or inconsistent primary care demand, inability to recruit GPs, and difficulty agreeing governance policies and streaming pathways. Despite concerns about GPs working outside the typical role of a GP, we found that where GPs are fully integrated in the ED work force there are perceived benefits to integrated working and managing the overall demand of the department [17].

Our findings are consistent with previous research where distinct primary care services were perceived as more open to provider-induced demand and integrated services are thought less affected because they are less visible and not publicised [8]. We also identified that low or inconsistent primary care demand has led to services being implemented but not considered cost-effective or useful in terms of managing flow, especially where GPs focussed only on primary care patients. As in other studies, primary care streaming was perceived to be effective where undertaken by experienced nurses who followed clear guidance and used their own clinical judgement to make decisions [8, 10, 18, 19]. However, we identified barriers such as implementing streaming in a primary care service where there was low primary care demand and challenges with aligning governance procedures which provided fewer opportunities for GPs to collaborate with ED staff.

#### Implications for policy and practice

Our findings have shown that implementing a health policy and model of service based on evidence from an exemplar model is not always feasible. Different models of service are necessary based on local contextual circumstances. Where the service operated as a separate primary care service in or alongside an ED, our findings show that it is important to consider challenges with GP recruitment, primary care demand, space in the department to locate GPs, streaming methods and compatible governance policies and procedures that support a culture of collaboration [3]. Where the service operated as an emergency medicine service then primary care demand might be less important, streaming is not essential, and GPs ‘location and access to investigations can be more flexible.

#### Future research

Our findings highlight the importance of a range of local and contextual factors that need to be explored with further research before recommending future policy changes. To support quality improvement further evaluation is needed to explore differences in outcomes (waiting times, patient flow, use of investigations, and admissions) across different models of primary care services in or alongside emergency departments. Further research is needed to evaluate the effectiveness of streaming and triage in EDs with primary care services and to understand their impact on patient flow and guide quality improvement, and challenges in funding such models. Finally, more research is needed to understand whether or how primary care models assist with the principal mission of EDs to treat patients with high acuity problems, or whether they may detract from this mission by attracting more low acuity patients (termed “provider-induced demand”) and generating overcrowding in emergency departments with other unintended consequences such as detriment to patient safety.

## Conclusion

Our study has highlighted how a health policy with a clear stated intended model has been challenging to implement and a “one size” approach does not fit all emergency departments. The ability to recruit and retain GPs, sustainable funding, clear governance frameworks, training, support and guidance and the physical space and layout of the department are important factors that should be considered when setting up a primary care service in or alongside an ED. Policy-makers, commissioners, managers and clinical leads need to consider how to employ GPs in different roles to meet variation in these factors. Local commissioners, ED and primary care services need to consider how primary care demand is defined and recognised and to what extent it is present in the ED. If there is a sufficient proportion of patients with primary care type problems, then streaming to a separate primary care service may be feasible. If the proportion of patients needing primary care is low, then integrating GPs into an emergency department where they can use their expertise more flexibly may also be viable.

## Supplementary information

**Additional file 1.** Key informant interview questions.

## Data Availability

The data that support the findings of this study are available from the corresponding author upon reasonable request.

## Abbreviations

ED: Emergency Department
GP: General Practitioner
NHS: National Health Service
NIHR: National Institute of Health Research
HS&DR: Health Service and Delivery Research Programme
