# Supplementary material for: Emergency department clinical leads’ experiences of implementing primary care services where GPs work in or alongside emergency departments in the UK: a qualitative study
Source: BMC Emerg Med. 2020 Aug 14;20:62. doi: 10.1186/s12873-020-00358-3 (PMC7429882; doi:10.1186/s12873-020-00358-3)
Supplement: Supplementary file 1 — Additional file 1. Key informant interview questions. [file 12873_2020_358_MOESM1_ESM.docx]

**Key informant interview questions**

**Hospital: Interview only**

**Clinical Director:**

**Survey respondent: Telephone contact:**

**GP-ED Model:**

**Date GPs started working in the department:**

**Reason for selection:**

1. **Department level – Demand/Flow**

| **Question** |
| --- |
| - In the survey you say that you have GPs working in a separate area next to the ED with a separate entrance, is that right? - How is the place where the GPs work labelled? Is it labelled as an urgent care centre? It is known by the public that there are GPs working there? |
| - So an ED nurse selects patients to be seen by a GP using locally developed inclusion criteria, can you explain more about how that works? - Is there a triage system or streaming system whereby the nurse can stream the patient to a GP or another member of staff? What are the options? Can you explain how the streaming works? - And there is a telephone triage where primary care service can make an appointment, can you explain how that works to me? - And patients can walk in and be seen? - So patients who need urgent care could be seen by a GP, ANP, ENP or Paramedic ACP, is that right? |
| - How does having GPs in your department affect the flow of patients in the ED? - Is there any exchange of patients from the GP area to the ED area and vice versa? |
| - You mentioned a home visiting service provided by advance care paramedics, can you explain more about how that works? |
| - The literature suggests that when people know that there are GPs working in the ED that they are more likely to go to the ED and so there is a provider induced demand. - We think that provider induced demand might occur more where there are separate areas where it is know that GPs work from as opposed to EDs where the GPs are embedded into the ED. Do you think that happens here? |
| - In the survey you say that there is also a GP out of hours service located at the hospital, is this a separate service to the GP service next to the ED (or the same)? - Were they previously separate and have now merged? - You mentioned in the survey something about the contract for OOH being renewed to include a 24/7 GP presence, 24 hour walk in and adjacent to the ED. Can you explain more about that? |

1. **Department level - Meeting the aim of GP service**

| **Question** |
| --- |
| - The literature suggests that GPs might admit less patients and do less investigations for some patients than a junior doctor for example. We have also found this talking to some other departments? - Are the GPs able to admit patients? - Why do you think that having GPs has not reduced hospital admissions in your department? |
| - You also said that most of the time there are improvements in quality of care given to certain types of cases |
| - You said that the GP service enables better use of available ED resources. Can you explain more about this? |
| - You also said that the GP service is never cost saving, why is this? |

1. **Department level- Changes in the service**

| **Question** |
| --- |
| - So you said a change of contract helped with setting up the service. So the contract is now held by the acute trust - What were the previous arrangements - previously contracts held separately for OOH and walk in by pseudo private and private providers |
| - You have said that your ED is making a capital bid, what does that involve? |

1. **Practitioner level questions**

| **Question** |
| --- |
| - You say that in addition to primary care and low acuity type patients - We notice that GPs have access to mainly tests that can be accessed in primary care and can order x-rays. Do patients who need X-rays remain in the urgent care centre or are they passed over to the ED? |
| - Another hospital has told us that the biggest advantages of having GPs working in the ED and not close by is the exchange of patients and that GPs and ED clinicians have the opportunity to seek advice from each other? Does this happen in your department? - Are there any learning experiences for GPs and ED doctors from working in a department with an urgent care centre next to it? |
| - We know from what other hospitals have said that there are problems recruiting GPs with the appropriate skillset to work in the ED. - Do the GPs working in the department there need to have a different skillset from GPs that might work in a local surgery? Might they see more,minor injuries or sicker patients? |
| - Do your junior doctors in the ED get to see patients with primary care problems? - Do you think they get to see enough of these patients? |
| - Have there been any issues with recruiting and staffing with GPs? We know in some areas it has been difficult to recruit GPs to work in the ED and sometimes a specific skillset is needed which can be difficult to meet. |

1. **Patient safety**

| **Question** |
| --- |
| - Do you think there are any positive or negative safety implications related with having GPs working in the ED? |
| - Some hospitals have reported rare cases of missed diagnoses from GPs seeing patients in the emergency department - are you aware of any such events? Were any changes made as a result to minimise future events? |
| - The literature suggests that having GPs working in the ED mean that the ED doctors can see more seriously unwell patients quicker, is that the case here? |

1. **Wider system level**

| **Question** |
| --- |
| - Do you think that having GPs in the ED produces local competition for local jobs? - If yes, then does that then make it difficult to for primary care to manage the demand in their service? |
| - Do you have any types of patients that visit the ED with primary care problems that might have problems accessing traditional primary care? For example, tourists, migrants, vulnerable groups |
